# Comparison of Phytolith Characteristics of Three Bamboo Species’ Cotyledon Organs

**DOI:** 10.3390/plants14081174

**Published:** 2025-04-10

**Authors:** Guomi Luo, Chengyao Liu, Rui Xu, Changming Wang, Taiyang Zhao, Mengsi Duan, Kemei Gao

**Affiliations:** 1 College of Forestry, Southwest Forestry University, Kunming 650224, China; 2023luoguomi@swfu.edu.cn (G.L.);; 2 Key Laboratory of Conservation and Utilization of Southwest Mountain Forest Resources, Ministry of Education, Kunming 650224, China; 3 Key Laboratory for Sympodial Bamboo Research, Southwest Forestry University, Kunming 650224, China

**Keywords:** bamboo, leaf, phytolith, Xishuangbanna

## Abstract

Phytoliths are widely used in plant taxonomy, paleoecology, soil silicon cycling, and agricultural archaeology. Bamboo has a strong capacity for silicon absorption, and there are some phytoliths in various organs. In this paper, the leaf organs (leaf blade, leaf sheath, culm sheath, and culm blade) of three kinds of bamboos [*B. vulgaris (Bambusa vulgaris*), *B. tulda* (*Bambusa tulda*), and *B. dolichoclada* (*Bambusa dolichoclada*)] were studied; the content, concentration, particle size distribution, and morphological characteristics of phytoliths in leaf organs were analyzed to explore the differences of phytoliths in different functional leaf organs of bamboo. The results showed that the content and concentration of phytoliths were the highest in the leaf sheath and the lowest in the culm sheath, and the content and concentration of phytoliths in the leaf blade and culm blade were between them. For different bamboo species, the order of phytolith content was *B. dolichoclada* > *B. tulda* > *B. vulgaris*, while the phytolith concentration was the opposite. The highest values of phytolith particle size peak distribution in the leaf sheaths were the opposite to those in leaf blades, culm sheaths, and culm blades. The particle sizes of phytoliths of the three bamboo species showed a similar trend. Only a few larger phytolith particle sizes were distributed in leaf blades and culm blades when they were larger than 400 μm. A total of 25 types of phytoliths were identified, and the leaf sheaths were mainly elongate and rondel phytoliths. The leaf blades are mainly saddle and rondel phytoliths with a unique phytolith morphology. Culm sheaths are dominated by rondel, scrobiculate, and acute phytoliths. Culm blades are similar to leaf blades but have a characteristic phytolith morphology. In addition, saddle phytoliths were the most abundant in the leaf blade and the least in the culm sheath, while rondel phytoliths were the most abundant in the culm blade and the least in the leaf sheath. The parameters of long-saddle phytoliths are different in different bamboo species and leaf organs. According to the long saddle phytolith parameters of different bamboo species, different bamboo species can be quantitatively distinguished to a certain extent. Therefore, this study not only helps to understand the differences in phytoliths in different bamboo species and leaf organs, but also provides a theoretical basis for bamboo species classification.

## 1. Introduction

Silicon (Si) is one of the essential elements for the growth and development of bamboo plants, and it is also the second most abundant element after oxygen in the Earth’s crust and soil [1,2]. Plants absorb the silicon in the soil through the roots, and deposit the silicon particles in the form of amorphous silicon dioxide in the plant cell wall or cell cavity, which is the “Phytolith” [3,4]. Phytoliths can not only provide direct, high-resolution records of vegetation and environmental changes on a short time scale [5], but also can be stably preserved in plant residues or soil for a long time; because of their morphological diversity and species specificity, they are widely used in plant classification, paleovegetation, soil silicon cycle research, and agricultural archaeology [6,7]. Liu et al.’s [8] study of typical plant communities in the mid-subtropics showed that phytoliths can distinguish herbaceous, shrub-herb, and forest communities. Morphological analysis of saddle-shaped phytoliths of 64 species of 19 genera in Bambusoideae showed that the plants of Bambusoideae could be distinguished by discriminant analysis [9]. In addition, the phytolith contents in different organs of the same plant are obviously different [10,11]. For example, Wang et al. [12] found that the phytolith content in leaves is much higher than that in culms and roots. Li et al. [13] found that the phytolith content of rice was sheaths > leaves > culms > spikes. As an important group of monocots, bamboo not only has high silicon accumulation capacity, but also its phytolith characteristics play an important role in plant function and ecological adaptation. It is an important object for the study of bamboo classification and discussion of environmental adaptability research [14,15].

The leaf organs of bamboo include vegetative and culm leaves. The vegetative leaves are composed of the leaf blade, leaf sheath, and petiole. The culm leaves are mainly composed of culm sheath and culm blade, and the petiole is an important characteristic that distinguishes bamboo from other herbaceous plants [16]. In this study, leaf sheath, leaf blade, culm sheath, and culm blade were selected as the research objects, and since the culm leaves have the same basic composition as the vegetative leaves, and the upper part of the culm sheaths have elongated leaves, the culm leaves are considered to be a special form of leaves or a stage in their evolution [17]. Culm leaves are usually regarded as a special structure to protect shoots or young shoots from injury, and there are obvious interspecific differences in culm leaves [17,18]. Previous studies have shown that there are significant differences in phytolith content, concentration, and morphology among different bamboo species and their organs, which are closely related to plant classification, function, and ecological adaptation [19]. For example, phytoliths in leaves are mostly used for auxiliary functions of photosynthesis and transpiration regulation, whereas phytoliths in leaf sheaths and culm sheaths are more associated with protection and mechanical support [20]. However, the distribution, particle size, and morphological characteristics of phytoliths in different bamboo species and their main organs have not been systematically studied. In particular, the distribution of phytoliths in different bamboo leaf organs in tropical regions, and the use of phytolith morphology for plant species classification and ecological significance, are still less studied.

*B. vulgaris* (*Bambusa vulgaris*), *B. tulda* (*Bambusa tulda*), and *B. dolichoclada* (*Bambusa dolichoclada*) are typical sympodial bamboos growing in southwest China. They belong to *Bambusa* of Poaceae, and grow in warm and humid climates [21]. However, there were great differences in the appearance of the three bamboo species, such as the color of the culm, the length of the culm, and the height of the bulge of the culm ring. In addition, the appearances of leaf organs are also different. The leaves of *B. vulgaris* are larger, and the culm leaves are harder and darker. *B. tulda* has relatively small leaves and thin culm leaves, usually pale or yellowish. *B. dolichoclada* is between the two [21,22,23,24,25]. Therefore, for three common bamboo species, *B. vulgaris*, *B. tulda*, and *B. dolichoclada*, at the Chinese Academy of Sciences in Xishuangbanna Tropical Botanical Garden, samples of the leaf blade, leaf sheath, culm sheath, and culm blade were collected. The phytolith content, concentration, particle size distribution, and morphological characteristics of different bamboo species and organs were systematically analyzed by using the improved wet oxidation method, combined with microscope observation and particle size analysis technology. In addition, the morphological parameters (length, width, and area) of long-saddle phytoliths were analyzed to explore the differences in phytolith characteristics of different bamboo species and organs and their ecological adaptation significance. This study aims to reveal the distribution and morphological characteristics of phytoliths in different organs of different bamboo species, and provide theoretical support and practical guidance for the classification, ecological function research, and silicon cycle mechanism of bamboo plants.

## 2. Results

### 2.1. Phytolith Content and Concentration Characteristics

From Table 1 and Table 2, it can be seen that the content and concentration of phytoliths in leaf sheaths were higher in different organs, but there were significant differences among bamboo species. The content of leaf sheaths in *B. tulda* (*Bambusa tulda*) and *B. dolichoclada* (*Bambusa dolichoclada*) was higher than 79 g/kg, and the leaf sheath phytolith concentration of *B. vulgaris* was the highest (32.26 × 10^6^ particles g^−1^). The phytolith content (96.35 g/kg) and phytolith concentration (23.61 × 10^6^ particles g^−1^) in culm blade of *B. dolichoclada* were significantly higher than those of other species. The content and concentration of culm sheath were significantly lower than those of other parts; in particular, the concentration of culm sheath in *B. dolichoclada* was the lowest (3.29 × 10^6^ particles g^−1^). Among different bamboo species, the phytolith content of *B. tulda* and *B. dolichoclada* was generally higher, and the culm blade of *B. dolichoclada* was the highest, while the leaf blade and leaf sheath of *B. tulda* were balanced. The phytolith content of *B. vulgaris* was the lowest, but the phytolith concentration of the leaf sheath was the highest. On the whole, the phytolith content was leaf sheath > culm blade > leaf blade > culm sheath, and the order of phytolith content was *B. dolichoclada* > *B. tulda* > *B. vulgaris* (*Bambusa vulgaris*). The order of phytolith concentration was leaf sheath > leaf blade > culm blade > culm sheath, but the phytolith concentration and phytolith content of different bamboo species were the opposite.

### 2.2. Phytolith Particle Size Characteristics

**Figure 1:**
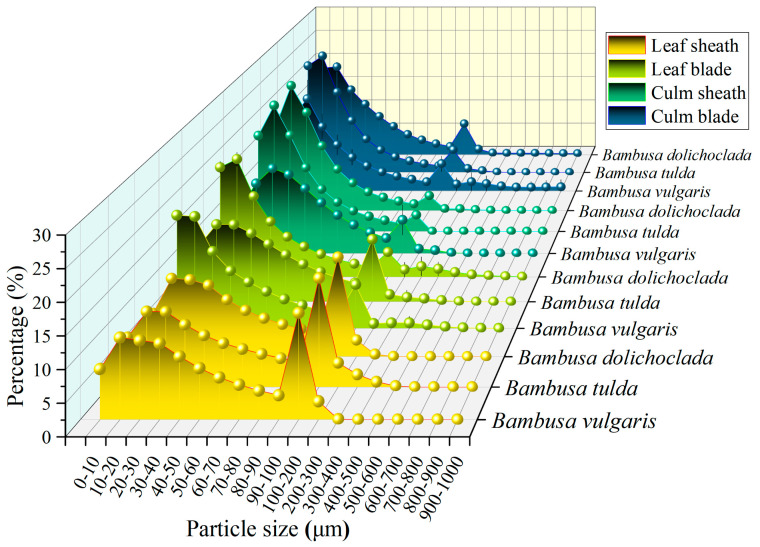
Phytolith particle size.

The particle size distribution of phytoliths was different in each bamboo species and organs (Figure 1). The maximum particle size peak distribution of phytoliths in leaf sheaths is 100–200 μm, the second maximum peak is 10–20 μm, and the proportion tends to zero when the phytoliths particle size is larger than 400 μm. The maximum particle size peak distribution of phytoliths in leaf blades, culm sheaths, and culm blades was 10–20 μm, followed by 10–200 μm. The percentage of phytoliths in leaf blades and culm blades was ≥0.01% when the particle size was 400–1000 μm. In addition, the particle size distribution proportion of phytoliths in leaf sheath, leaf blade, culm sheath, and culm blade is very small when they are larger than 200 μm. However, among the three bamboo species, phytolith particle size showed a similar trend.

### 2.3. Phytolith Morphology and Assemblages

#### 2.3.1. Phytolith Morphotypes

**Figure 2:**
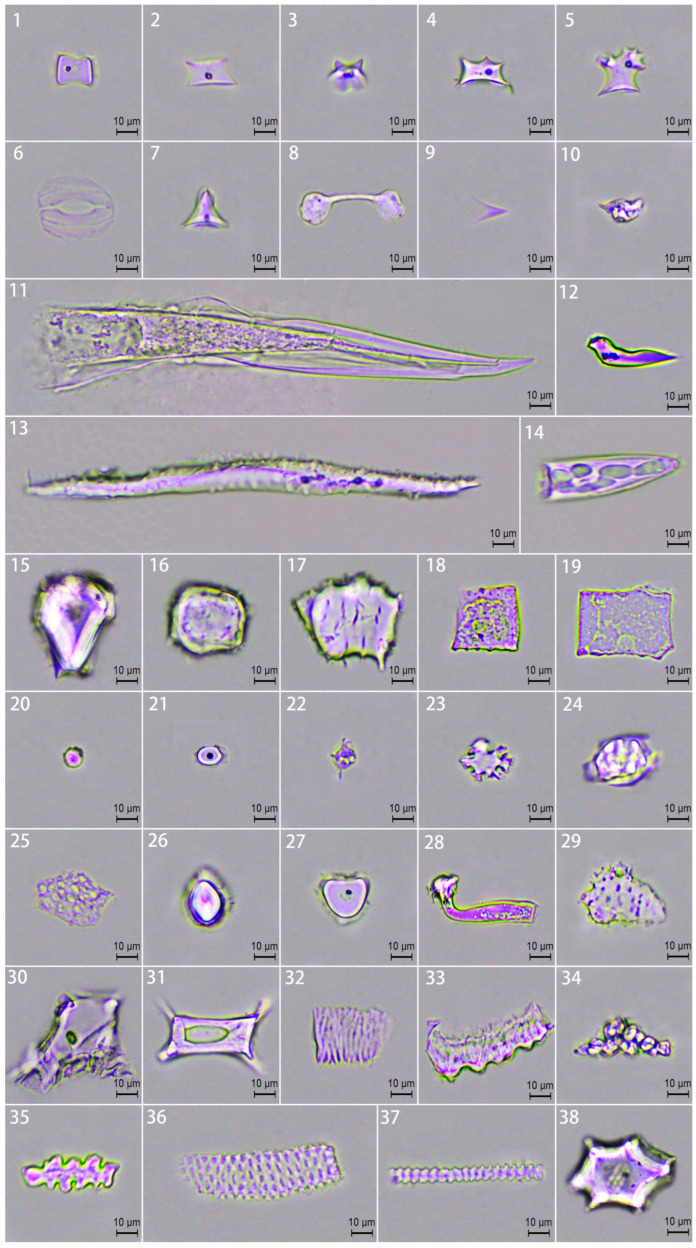

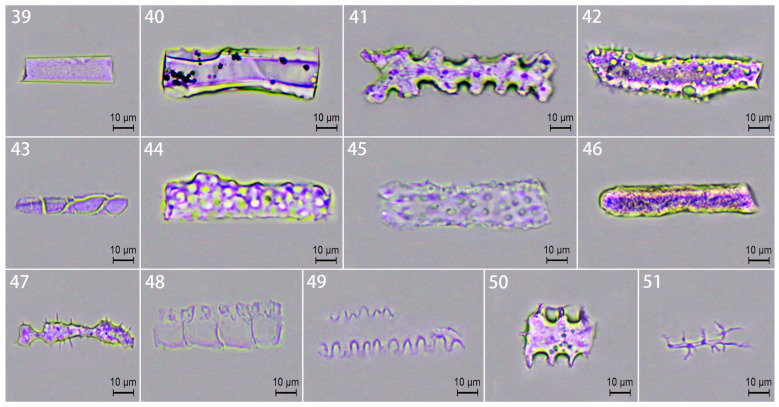
Phytolith morphology and assemblage. 1. Saddle; 2. Ruffle top rondel; 3. Two-spiked rondels; 4. Three-spiked rondels; 5. Irregula rondels; 6. Stomata; 7. Dumbbell; 8. Bilobate; 9–14. Acute; 15. Flabellate; 16–17. Bulliform; 18–19. Blocky; 20. Circular; 21. Oblong; 22. Circular baculate; 23. Papillate; 24. Circular tuberculate; 25. Scrobiculate; 26. Fusiform; 27. Scutiform; 28. Bulbous; 29. Baculate; 30. Conical; 31. Irregula blocky; 32. Tracheary; 33. Sinuate; 34. Epidermal phytoliths; 35. Lobate; 36. Scaly; 37. Carinate; 38. Polygonal; Elongate:39. Elongate entire; 40. Four-elongate prismatic; 41. Elongate cavate terminal; 42. Elongate hollow; 43. Elongate rift; 44. Elongate papillar; 45. Elongate scrobiculate; 46. Three-elongate prismatic; 47. Elongate baculate; 48. Elongate gibbate; 49. Elongate sinuate; 50. Elongate dentate; 51. Dendritic.

According to the international classification standards of ICPN 1.0 and ICPN 2.0 [6,26], the leaf sheath, leaf blade (including the petiole), culm sheath, and culm blade phytoliths of three bamboo species were identified and statistically analyzed; a total of 25 phytoliths were identified (Figure 2). The main phytoliths are saddle (long-saddle, short-saddle), rondel (ruffle top rondel, two-spiked rondels, three-spiked rondels, irregula rondels), stomata, dumbbell, bilobate, acute, flabellate, bulliform, blocky, circular (circular entire, oblong, circular baculate, circular tuberculate), scrobiculate, fusiform, scutiform, bulbous, baculate, conical, tracheary, sinuate, epidermal phytoliths, lobate, scaly, carinate, polygonal, elongate, and dendritic. In addition, the types of phytoliths in the culm sheath were numerous, and the types of phytoliths in the leaf sheath, leaf blade, and culm blade were relatively uniform. The common phytoliths are saddle, rondel, stomata, bilobate, acute, bulliform, circular, scrobiculate, bulbous baculate, epidermal phytoliths, elongate, bulbous, and baculate. Specific phytoliths are found only in leaf blade (lobate and polygonal) and culm blade (scaly and carinate).

#### 2.3.2. Phytolith Assemblages

**Figure 3:**
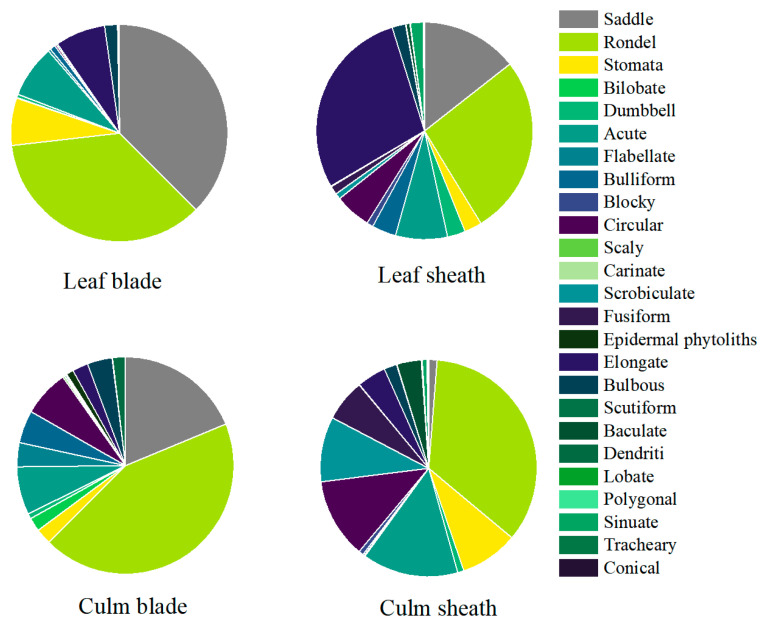
Changes in phytolith assemblages in different leaf organs.

According to the assemblage characteristics of phytoliths in three different bamboos and their organs (Table 3 and Figure 3), the assemblage characteristics of phytoliths among different bamboos and their leaf organs are obviously different.

In particular, the frequency of rondel phytoliths in the leaf organs of the three bamboo species was high. The proportion of saddle phytoliths in culm sheath was lower than that in leaf blade, and the proportion of elongate and dumbbell phytoliths was the highest in leaf sheath, but the proportion of dumbbell phytoliths was less than 5%. In addition, the proportion of stomata phytoliths in *B. vulgaris* (*Bambusa vulgaris*) leaf blades was as high as 14.08%, which was 1.33 times of culm sheath, 3.78 times of culm blade, and 8.33 times of leaf sheath. Acute phytoliths were up to 14.00% in culm blades, 1.27 times in culm sheaths, 2.12 times in leaf blades, and 2.06 times in leaf sheaths. Bulliform phytoliths were up to 11.23% in leaf sheaths, 13.86 times in leaf blades, 1.56 times in culm blades, and 16.28 times in culm sheaths. The proportions of circular and scrobiculate phytoliths in culm sheaths were as high as 18.35% and 24.31%, which were the highest among the three bamboo species. The proportion of fusiform phytoliths in *B. tulda* (*Bambusa tulda*) culm sheaths was as high as 21.22%; no fusiform phytoliths were found in *B. tulda* other leaf organs. Circular phytoliths were 8.1% in culm blades, 26.13 times in leaf blades, 1.89 times in leaf sheaths, and 8.1 times in culm sheaths. The proportion of acute phytoliths in leaf sheath was 6.75%, and that of other organs was less than 5%. In *B. dolichoclada* (*Bambusa dolichoclada*), the proportion of acute phytoliths in culm sheaths was as high as 24.19%, which was 1.54 times of that in leaf blades, 2.37 times of that in leaf sheaths, and 3.41 times of that in culm blades. The proportion of circular phytoliths in culm sheath is 14.86%, which is 3.23 times of that in leaf sheath and 1.45 times of that in culm blade. In contrast, the proportion of other phytoliths in the leaf organs of the three bamboo species was relatively low, and there were also some differences (Table 3).

In addition, we used the observed 25 phytolith morphologies to analyze their variations in the leaf blade, leaf sheath, culm sheath, and culm blade, which showed some differences in morphology (Figure 3). The proportion of rondel phytoliths in leaf organs was the highest, followed by culm blade > leaf blade > leaf sheath > culm sheath. Nine types of phytolith morphology (rondel, bilobate, flabellate, bulliform, circular, epidermal phytoliths, bulbous, dendritic) were found in the culm blade with the highest proportion and varied with the organs. On the contrary, five phytolith morphologies (stomata, acute, scrobiculate, elongate, sinuate) had the lowest proportion in the culm blade. Three types of phytolith morphology (baculate, fusiform, dumbbell) were found in less than 1% of the culm blade. Eight types of phytolith morphology (stomata, acute, circular, scrobiculate, fusiform, scutiform, baculate, tracheary) had the highest proportion in culm sheath. In contrast, the six phytolith morphologies (saddle, flabellate, bulliform, blocky, bulbous, conical) had the lowest proportion in the culm sheath. The proportion of bilobate was about 1%, the proportion of rondel phytoliths was higher than that of leaf sheath, and the types of phytolith morphology were the most abundant. The proportion of three phytolith forms (saddle, lobate, polygonal) in the leaf blade was the highest. In contrast, seven phytolith morphologies (bilobate, dumbbell, circular, epidermal phytoliths, baculate, sinuate, and tracheary) were least abundant in the leaf blade. The morphology of other phytoliths is lower than that of the culm blade, culm sheath, and leaf sheath. Five types of phytoliths (dumbbell, blocky, elongate, sinuate, conical) were most abundant in leaf sheaths. In contrast, three phytolith morphologies (rondel, scutiform, dendritic) were least abundant in leaf sheaths.

#### 2.3.3. Long-Saddle Phytolith Parameters

**Figure 4:**
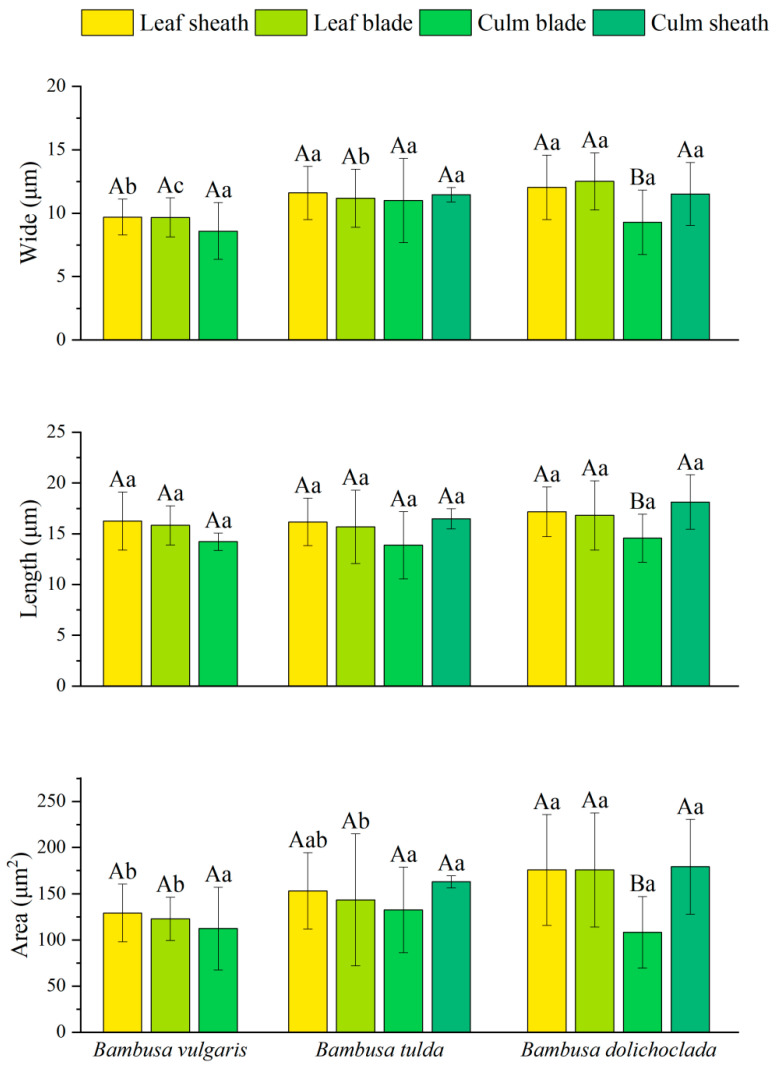
Long-saddle phytolith parameters. Note: different capital letters indicate significant differences between different organs of the same bamboo species (*p* < 0.05), and different lower case letters indicate significant differences between the same organ of different bamboo species (*p* < 0.05).

As can be seen from Figure 4, the width, length, and area of long-saddle phytoliths varied among bamboos or organs (*p* < 0.05). Among them, the length, width, and area of long-saddle phytoliths in the leaf sheath are generally higher than those in other organs, and the length of long-saddle phytoliths in the culm blade is the smallest. For the same bamboo species, only the length, width, and area of the *B. dolichoclada* (*Bambusa dolichoclada*) culm blade were significantly different from those of long-saddle phytoliths in other organs, while there were no significant differences between the other two species. For the same organ, the long-saddle width of the leaf blade was significantly different among different bamboo species. The leaf sheath of *B. vulgaris* (*Bambusa vulgaris*) was significantly lower than that of *B. dolichoclada* and *B. tulda* (*Bambusa tulda*), and the long-saddle width of the culm sheath and culm blade were not significant. In addition, the long-saddle phytolith length of each organ was not significant among different bamboo species. The long-saddle phytolith area of *B. dolichoclada* showed a significant advantage among the leaf sheath, leaf blade, and culm sheath, but the long-saddle phytolith area of the culm blade was the lowest among all bamboo species. In general, the long-saddle phytolith parameters of *B. vulgaris* are smaller than those of *B. tulda* and *B. dolichoclada*.

**Figure 5:**
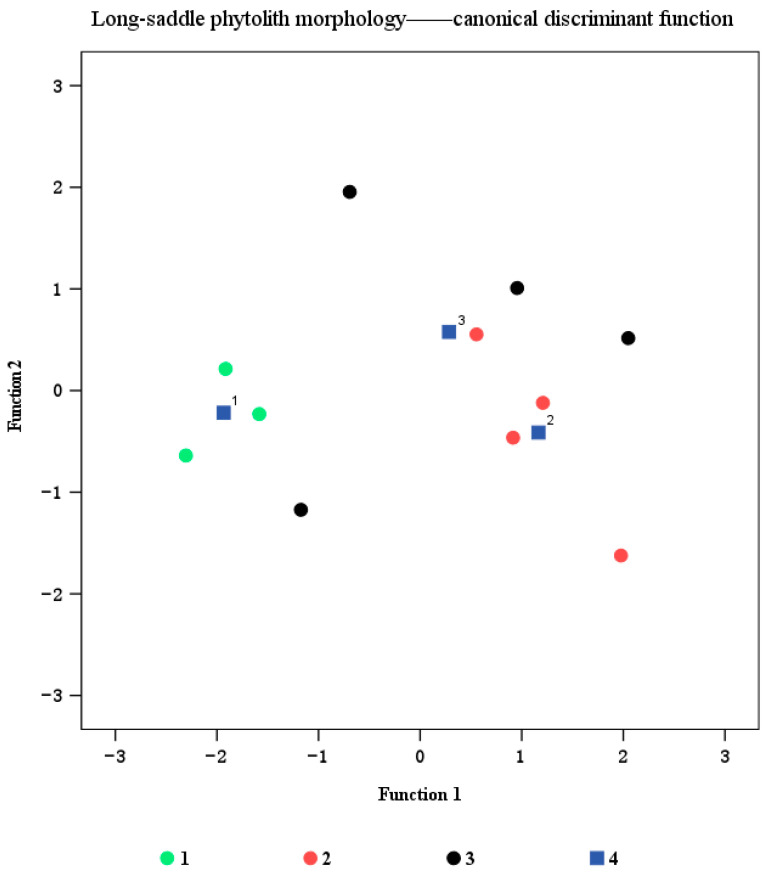
Scatter plot of morphological parameters of long-saddle phytoliths in three bamboo species. The symbols in the graph represent 1. *Bambusa vulgaris*; 2. *Bambusa tulda*; 3. *Bambusa dolichoclada*; 4. Position of center of gravity of each category.

In order to quantitatively characterize the differences in phytoliths of three bamboo species, discriminant analysis was used to distinguish three bamboo species based on long-saddle phytolith parameters. The results showed that 72.7% of the samples could be correctly classified by long-saddle phytolith parameters. Among them, the classification accuracy of *B. vulgaris* is the highest, reaching 100%, followed by *B. tulda* up to 75%, and the lowest is *B. dolichoclada*, reaching 50%. Therefore, from the perspective of bamboo species, the discriminant function of different bamboo species constructed by long-saddle phytolith parameters can distinguish different bamboo species to a certain extent. In particular, *B. vulgaris* and *B. tulda* had better results (Figure 5 and Table 4).

## 3. Discussion

### 3.1. Variation in Phytolith Content and Concentration in Different Organs of Different Bamboo Species

Phytoliths have a variety of functions in plants, such as structural support, disease defense, and reducing transpiration and water loss [28]. Due to the difference in transpiration intensity in different organs of plants and the difference of plants, the phytolith content of plants or different organs of the same plant will be different [29,30,31]. Studies have shown that poaceae plants are one of the groups with high phytolith content, especially bamboo plants. The content and concentration of phytoliths in bamboo vary significantly among species and organs [32]. With the increase in temperature and transpiration rate, the silicon content and phytolith content were the highest at the end of the shooting stage, and gradually decreased during the dormant period [33,34]. It is also influenced by the proportion of cell types during plant growth [34]. Previous studies have shown that the phenomenon of plant water spitting is an important indicator of the vigorous physiological activities of plant roots [35]. The *Zea mays* and the *Phragmites australis* also have the water spitting phenomenon, in the different growth periods, and spit the water at night differently [34,36,37]. *B. vulgaris* (*Bambusa vulgaris*), *B. tulda* (*Bambusa tulda*), and *B. dolichoclada* (*Bambusa dolichoclada*) belong to the poaceae family with *Z. mays* and *P. australis*, and the silicon in the plant is transported to the plant with water. This may be responsible for the uneven distribution of phytolith concentrations in the organs of *B. vulgaris*, *B. tulda*, and *B. dolichoclada*. It was found that the phytolith concentration of *Phragmites australis* reached a peak in August and a valley in September. It was suggested that the variation in phytolith concentration may be consistent with the requirement of silicon for plant growth [34]. In the present study, both phytolith content and concentration were significantly higher in the leaf sheath than in the culm sheath (Table 1 and Table 2), which is similar to the results of Li’s study on *Oryza sativa* [38]. In contrast, the culm sheath had the lowest phytolith content and concentration (Table 1 and Table 2), which may be related to the fact that the culm sheath mainly provides protection at the initial stage of bamboo shoots, and then gradually falls off; therefore, high phytolith content may not be needed to provide additional protection or structural support [24,38,39,40,41]; this is similar to the results of Liu et al. [34] on *Phragmites australis* in northeast China and Zhu et al. [33] on the phytoliths of *Dendrocalamus giganteus* in different phenological periods. Compared with the study by Hodson et al. [40], this study further quantified the differences in phytolith concentrations in different bamboo species and their organs. The results showed that the phytolith content and concentration in the leaf blade and leaf sheath were relatively balanced (Table 1 and Table 2), which may indicate that they had strong adaptability to different growth environments. This finding supports the hypothesis of phytoliths as indicators of adaptive function [28].

### 3.2. Functional Relationships Between Phytolith Morphology and Proportion in Bamboo Leaf Organs

Phytoliths are biomarkers of plant adaptation to the environment. The morphology, type, and distribution of phytoliths are controlled by the shape and size of plant cells and intercellular spaces; plant cells and intercellular spaces are also closely related to water, temperature, soil type, and other environmental stresses [42,43,44,45,46,47]. A total of 25 phytoliths were identified in the leaf sheath, leaf blade, culm sheath, and culm blade of the three bamboo species, and significant differences in phytolith morphology and distribution were found (Figure 2); these differences may be due to the heterogeneity of plant cell structure [46]. The anatomical study of Bambusoideae leaves by Motomura et al. [48] showed that phytolith deposition in leaves is not uniform and that spindle cells in mesophyll tissues are preferentially silicified (especially at the maturity stage); while chloroplast-containing parenchyma cells do not participate in silicification, short cells and bulliform cells (such as saddle and dumbbell precursor cells) in epidermal cells are the first to deposit silicification [49], and the silicification degree of other epidermal cells increased with leaf age. The degree of silicification was also different in different growing years and environments [50,51]. In this study, the proportion of rondel and saddle phytoliths was higher in the leaf blades, leaf sheaths, and culm blades of the three bamboo species. The proportion of saddle phytoliths in culm sheaths was significantly lower than that in other leaf organs, but the proportion of scrobiculate and acute phytoliths was higher than that in other leaf organs (Figure 2 and Figure 3, Table 3). This suggests that the culm sheath epidermis may lack short cells and bulliform cells (such as precursor cells of bilobate and saddle phytoliths), which are regularly arranged in the leaf blades, and may be dominated by long cells or unspecialized cells, in order to help bamboo resist external damage so as to adapt to the environment. This is similar to the results of Xu et al. [50] and Jin et al. [51] on *Ferrocalamus strictus* strictus and six bamboo species. Obon and Rivera [52] suggest that vascular long cells, including xylem and phloem, are mainly elongate phytoliths involved in water conduction, nutrient storage, and mechanical support in plants. The increase in elongate phytoliths plays an important role in plant support [53]. In this study, the elongate phytoliths were mainly formed in the leaf sheath, and the proportion was higher than that in the leaf blade, culm sheath, and culm blade (Figure 2 and Figure 3, Table 3). This illustrates that phytolith assemblages in leaf tissue are affected to some extent by cell physiology and its climate change, which is similar to the findings of Li et al. in *Dendrocalamus* leaves [54]. Culm sheaths and culm blades mainly protect young culms temporarily and regulate water exchange, with less mechanical requirement. As the main site of photosynthesis, the silicification strategy of leaves is more focused on maintaining gas exchange efficiency. The leaf sheath affects the water state of leaf blades to a certain extent, and has auxiliary regulation of the transpiration of leaf blades, which may also result in more elongate phytoliths in the leaf apparatus [51,55,56]. This study also found that the distribution of phytolith types varied with leaf organs among different bamboo species (Figure 2 and Figure 3, Table 3), which may be closely related to the growth environment and ecological adaptation strategies of different bamboo species [42]. Wang et al. showed similar results for *P. edulisamg* and two variants in culm leaf phytoliths [57].

### 3.3. Ecological Significance of Significant Differences in Phytoliths Among Different Bamboo Species and Organs

Related studies have shown that the proportion of dominant phytoliths in leaves, such as round-ended bilobate, flat-ended bilobate, and saddle phytoliths, can provide a reference for the classification of bamboo. In addition, different bamboo species have their own special types of phytoliths, and these characteristics can be used to classify and identify bamboo [9,58]. Analysis of long-saddle phytoliths from 64 bamboo species belonging to 19 genera showed that the length and width of long-saddle phytoliths were 15.00–21 μm and 9.0–15.0 μm, respectively [9]. Tao et al. researched 17 species of bamboo in three genera, the average length and width of the oblong concave saddles (long-saddle) were 19.2 μm and 10.5 μm in *Phyllostachys*, 18.00 μm and 8.7 μm in *Dendrocalamus*, and 17.2 μm and 9.4 μm in *Bambusa*, respectively [59]. In this study, the length and width of long-saddle phytoliths in different organs of bamboo were within this range (Figure 4), which supported the above research. In the present study, the area of long-saddle phytoliths in leaf sheaths was generally higher than that in leaf blades, culm sheaths, and culm blades (Figure 4), indicating that leaf sheaths may require stronger mechanical support functions to cope with environmental stresses, and that leaf sheaths may be more sensitive to environmental stresses, especially in resistance to wind, pests, and mechanical damage [60,61]. He et al. showed similar results for *Bambusa emeiensis* phytoliths [26]. In addition, the leaf sheath of bamboo is a key region for plant water regulation and defense mechanisms, and the large area of long-saddle phytoliths may contribute to enhancing this function [60]. The long-saddle phytoliths of the culm blade are relatively small (Figure 4), probably because their main function is to support plant growth and branching rather than defense or water regulation [49]. Therefore, the development of culm blades in long-saddle phytoliths may be limited by their structural requirements. Previous studies have shown that the differences among bamboo species are not only reflected in the overall morphology, but also at the micro level of cells and tissues [61]. In this study, for the same bamboo species, there were significant differences in length, width, and area between culm blades of *B. dolichoclada* (*Bambusa dolichoclada*) and long-saddle phytoliths of other organs, which indicates that different bamboo species have different morphological characteristics in similar organs, which supports the above research. The width of long-saddle phytoliths in the leaf blade was significantly different among different bamboo species, which may be related to the demand for photosynthesis and water regulation during their growth and development [62]. In terms of long-saddle phytolith length, this study showed no significant differences among different bamboo species, which may be due to the fact that bamboo species are under similar genetic control in this dimension and environmental factors have little effect on it [63,64]. This study also found that the discriminant function of different bamboo species constructed by long-saddle phytolith parameters could distinguish different bamboo species to a certain extent (Figure 5 and Table 4). This may reflect the genetic differences of cell development among bamboo species and provide a stable microscopic index for classification. Although this study reveals the distribution and morphological characteristics of phytoliths in different bamboo species and organs, there are still some problems worthy of further discussion. For example, the mechanism of phytolith formation and its association with plant physiological functions are not fully understood. In the future, we will continue to combine molecular biology and cytological techniques to study the process of plant rock deposition in plant cells and its relationship with plant growth and development.

## 4. Materials and Methods

### 4.1. Regional Setting

Samples were collected from the Chinese Academy of Sciences Xishuangbanna Tropical Botanical Garden (101°25′ E, 21.41′ N, approximately 570 m above sea level), which is strongly influenced by the southwest monsoon and has a humid climate with an average annual temperature of 21.4 °C and annual precipitation of 1560 mm, with dry and wet seasons.

### 4.2. Sampling

**Figure 6:**
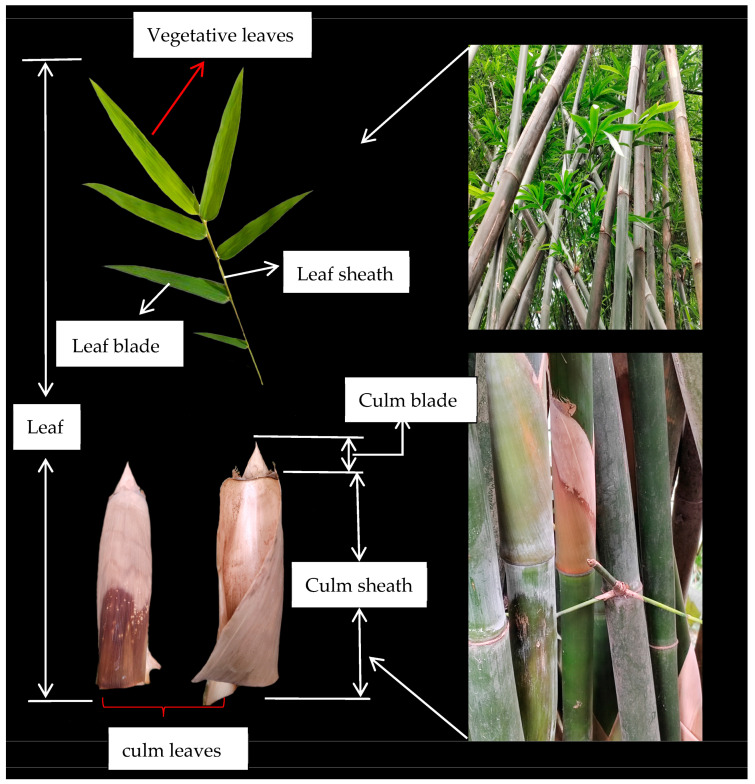
Instructions for bamboo sample collection (Take the *Bambusa dolichoclada* bamboo, for example). Note: In the figure: vegetative leaves include leaf sheath, leaf blade; culm leaves include culm sheath; culm blade.

Sampling was referred to the method from Jing et al. [51] and Wang et al. [57]. The sun-facing surfaces of mature leaf blades, leaf sheaths, culm sheaths, and culm blades of three annual bamboo species [*B. vulgaris* (*Bambusa vulgaris*), *B. tulda* (*Bambusa tulda*), and *B. dolichoclada* (*Bambusa dolichoclada*)] were sampled. The samples were taken from the middle of the bamboo. Six kinds of bamboo with similar diameter, height, sampling time, and location were chosen (collected in the fall of 2022). Among them, mature leaf blade: in each bamboo culm in the middle part, select the growth of robust, pest-free mature leaf blade for sampling. Use scissors to cut the entire leaf blade, including the petiole. At least 5 leaves of each species of bamboo were collected as samples and immediately put into a sealed bag, and the information of bamboo species, sampling date, and sampler was marked. Leaf sheath: the part of the leaf sheath attached to the leaf was collected at the same time as the leaf was cut. Make sure the leaf sheath is intact, and record its length, color, and texture characteristics. Leaf blade samples with leaf sheaths were stored together in a sealed bag. Culm sheath: Look for culm sheaths that have matured on the culm and are not falling off. Peel them from the middle of the culm. Care should be taken not to destroy the integrity of the culm sheath and to record its size, color, texture, etc. Culm blade: after the culm sheath is peeled, the exposed culm blade should also be sampled. Use scissors to carefully cut off the culm blade to avoid damaging its morphological structure. The number, size, and shape of culm blade segments were recorded and stored together with their corresponding culm sheath in sealed bags. Finally, the bagged sample was taken back to the laboratory and washed with purified water to remove sediment and dust. The sample was used for phytolith extraction (Figure 6).

### 4.3. Phytolith Extraction

Phytoliths were extracted by improved wet air oxidation method [65]. The sample was dried at 60 °C, and 0.3 g of the sample was weighed. After defoaming with 10 mL HNO_3_ and 2 mL HCLO_4_, the sample was digested by microwave for 2 h, and then 3 mL concentrated HCL was added to further remove impurities. The sample was cold-transferred to 14 mL centrifuge tube volume and centrifugally cleaned to neutral; spore tablets were added and dissolved with dilute HCL, and the sample was centrifugally cleaning again, then the bottom of the precipitation of white material was phytoliths. The fixing sheet was made of neutral resin, and more than 100 photos were taken under 40× Olympus BX53 microscope (Country of manufacture: China; Manufacturer: Ningbo Shunyu Instrument Co., Ltd.; Equipment source city: Yuyao City, China) for phytolith morphology analysis. Phytoliths are classified and described in accordance with the morphological classifications proposed by ICPN 1.0 and ICPN 2.0 [6,26].

Phytolith content (g/kg) = phytolith weight after drying (g)/sample weight (g) × 1000; phytolith concentration (particles/g) = (The number of phytolith particles was counted under microscope × 10,315)/(The number of lycopodium spores was counted by microscope × 0.3 g). Furthermore, the length, width, and area parameters of long-saddle phytoliths in different parts of each bamboo species were measured by ImageJ (Version number:1.54b) [57]. The particle size of phytoliths was measured by Malvern software (Version number: Mastersizer 3000 + Hydro EV). Each sample was measured 3 times.

### 4.4. Data Analysis

Trial data processing was performed using Excel 2016 and SPSS 27.0. One-way ANOVA was used to analyze the differences in phytolith content and long-saddle phytolith parameters. In the Origin 2021 software, the data were plotted, and Adobe Photoshop was used for phytolith morphology typesetting.

## 5. Conclusions

The phytolith characteristics of three different bamboo leaf organs were studied. The results showed that 1) phytolith contents in different organs were leaf sheath > culm blade > leaf blade > culm sheath. Phytolith concentrations in different organs were leaf sheath > leaf blade ≈ culm blade > culm sheath. In addition, the phytolith content of *B. tulda* and *B. dolichoclada* was much higher than that of *B. vulgaris*, while the phytolith concentration showed the opposite trend. The highest values of phytolith particle size peak of the leaf sheath were the opposite to those of the leaf blade, culm sheath, and culm blade. When the particle size of the phytolith was larger than 400 μm, only a few larger phytoliths were found in the leaf blades and culm blades. A total of 25 phytoliths were observed in three bamboo species and their leaf organs. There were significant differences in the morphology and proportion of different bamboo species and leaf organs. Among them, the leaf sheaths were mainly rondel and elongate phytoliths. The leaf blades were mainly rondel and saddle phytoliths; lobate and polygonal phytoliths were found only in the leaf blades. Culm sheaths had the most diverse phytolith types, mainly rondel, scrobiculate, and acute phytoliths. Culm blades were similar to leaf blades, but scaly and carinate phytoliths occurred only in culm blades. The parameters of long-saddle phytoliths varied among different bamboo species and leaf organs. According to this, *B. vulgaris*, *B. tulda*, and *B. dolichoclada* can be identified to a certain extent. The phytolith parameters (length, width, area) of leaf sheaths were generally larger than those of other organs, while the phytolith parameters of the culm blade were the smallest. The phytolith parameters of *B. vulgaris* were lower than those of *B. tulda* and *B. dolichoclada*. In conclusion, this study not only helps to understand the distribution of phytoliths in different leaf organs of bamboo, but also provides a theoretical basis for the classification of bamboo plants.

## Figures and Tables

**Table 1:** Phytolith content.

Bamboo Species	Leaf Blade (g/kg)	Leaf Sheath (g/kg)	Culm Blade (g/kg)	Culm Sheath (g/kg)	Means (g/kg)
*Bambusa vulgaris*	23.22 ± 0.46 ^Ab^	43.13 ± 5.15 ^Ab^	37.06 ± 4.37 ^Ab^	13.17 ± 3.09 ^Cc^	29.15 ± 12.64 ^b^
*Bambusa tulda*	70.54 ± 10.44 ^Aa^	81.99 ± 11.55 ^Aa^	74.92 ± 4.17 ^Aa^	37.07 ± 4.19 ^Ba^	66.13 ± 19.38 ^a^
*Bambusa dolichoclada*	63.08 ± 23.71 ^Aab^	79.69 ± 9.58 ^Aa^	96.35 ± 19.29 ^Aa^	29.96 ± 0.60 ^Cb^	67.27 ± 29.05 ^a^
Means (g/kg)	52.28 ± 19.99 ^A^	68.27 ± 20.48 ^A^	64.83 ± 34.09 ^A^	26.73 ± 10.94 ^B^	/

Note: different capital letters in the same row indicate significant difference among different organs (*p* < 0.05), and different lowercase letters in the same column indicate significant difference among different bamboo species (*p* < 0.05).

**Table 2:** Phytolith concentration.

Bamboo Species	Leaf Blade (×10^6^ Particles g^−1^)	Leaf Sheath (×10^6^ Particles g^−1^)	Culm Blade (×10^6^ Particles g^−1^)	Culm Sheath (×10^6^ Particles g^−1^)	Means (×10^6^ Particles g^−1^)
*Bambusa vulgaris*	18.73	32.26	13.86	5.33	17.56
*Bambusa tulda*	19.19	19.45	10.56	9.93	13.28
*Bambusa dolichoclada*	11.06	14.49	23.61	3.29	13.11
Means (×10^6^ particles g^−1^)	16.33	22.07	16.01	6.18	/

**Table 3:** Morphology and proportion of phytoliths in leaf organs of three bamboo species.

Phytolith Assemblage	*Bambusa vulgaris*				*Bambusa tulda*				*Bambusa dolichoclada*			
	Leaf Sheath (%)	Leaf Blade (%)	Culm Blade (%)	Culm Sheath (%)	Leaf Sheath (%)	Leaf Blade (%)	Culm Blade (%)	Culm Sheath (%)	Leaf Sheath (%)	Leaf Blade (%)	Culm Blade (%)	Culm Sheath (%)
Saddle	9.53	32.31	16.85	/	14.33	46.78	6.92	1.00	20.19	33.25	31.03	2.48
Rondel	23.52	37.91	43.11	25.00	35.91	30.81	60.67	36.16	17.27	38.62	28.9	41.9
Stomata	1.69	14.08	3.72	10.55	4.28	2.35	2.77	4.99	1.22	2.94	0.89	9.9
Bilobate	/	/	/	/	/	/	0.59	/	/	0.38	4.96	/
Dumbbell	1.06	0.36	0.44	0.69	4.12	0.72	1.38	1.75	2.43	0.38	0.53	0.57
Acute	6.78	6.59	14.00	11.01	6.75	2.97	0.99	4.99	10.22	15.73	7.09	24.19
Flabellate	/	0.09	0.22	0.23	/	0.82	0.59	0.25	/	0.26	9.04	/
Bulliform	11.23	0.81	7.22	0.69	/	0.92	5.14	/	0.24	0.64	2.66	/
Blocky	1.06	/	/	1.61	1.48	/	/	0.75	/	/	/	/
Circular	7.63	0.36	1.53	18.35	4.28	0.31	8.1	1.00	4.62	/	10.28	14.86
Scaly	/	/	/	/	/	/	/	/	/	/	0.71	/
Carinate	/	/	/	/	/	/	/	/	/	/	0.18	/
Scrobiculate	2.54	0.27	0.44	24.31	/	0.2	0.2	6.23	0.24	0.13	/	0.19
Fusiform	4.03	/	0.22	/	/	/	/	21.2	/	/	0.35	/
Epidermal phytoliths	/	0.09	3.5	0.23	/	/	/	/	0.24	/	/	/
Elongate	29.03	3.79	3.5	1.38	22.08	13.1	1.19	7.48	37.96	5.63	2.48	4.38
Bulbous	0.64	3.25	5.25	4.82	2.97	0.41	5.53	0.25	2.19	1.92	0.9	0.77
Scutiform	/	/	/	/	0.18	/	/	/	/	0	/	0.19
Baculate	/	/	/	0.69	1.48	/	0.2	11.47	/	0.12	/	/
Dendriti	0.21	/	/	0.44	/	/	5.73	/	/	/	/	/
Lobate	/	0.09	/	/	/	/	/	/	/	/	/	/
Polygonal	/	/	/	/	/	0.41	/	/	/	/	/	/
Sinuate	1.05	/	/	/	2.14	0.1	/	2.48	2.43	/	/	/
Tracheary	/	/	/	/	/	0.1	/	/	/	/	/	0.38
Conical	/	/	/	/	/	/	/	/	0.75	/	/	0.19
Types	15	13	13	14	12	14	13	13	13	12	14	12

**Table 4:** Discrimination results of three kinds of bamboos.

	Bamboo Species	Distinction Type			Total
		*Bambusa* *vulgaris*	*Bambusa tulda*	*Bambusa* *dolichoclada*	
Discriminant number	*Bambusa vulgaris*	3	0	0	3
	*Bambusa tulda*	0	3	1	4
	*Bambusa dolichoclada*	1	1	2	4
Correct rate (%)	*Bambusa vulgaris*	100.0	0.0	0	100.0
	*Bambusa tulda*	0.0	75.0	25.0	100.0
	*Bambusa dolichoclada*	25.0	25.0	50.0	100.0

## Data Availability

All data generated or analyzed during this study are included in the article.
